# A practical method for integrating community priorities in planning and implementing cancer control programs

**DOI:** 10.1007/s10552-023-01688-w

**Published:** 2023-04-18

**Authors:** Emily Bilenduke, Andrea J. Dwyer, Elsa S. Staples, Kristin Kilbourn, Patricia A. Valverde, Maria E. Fernández, Betsy C. Risendal

**Affiliations:** 1 Department of Psychology, University of Colorado Denver, Denver, CO USA; 2 Department of Community and Behavioral Health, School of Public Health, University of Colorado, Aurora, CO USA; 3 Center for Health Promotion and Prevention Research, University of Texas Health Science Center at Houston School of Public Health, Houston, TX USA

**Keywords:** Community-engaged research, Implementation mapping, Hereditary cancer screening, Community advisory board

## Abstract

**Purpose:**

Community engagement is essential in effective public health programs. This paper illustrates the methods used to engage community in the development of a multi-level implementation intervention to address cancer disparities related to hereditary cancer syndromes.

**Methods:**

Implementation Mapping (IM), was used to guide the co-creation of an intervention. Key partners were recruited to a 13-member statewide community advisory board (CAB) representing healthcare and community-based organizations. As part of a needs assessment, a 3-round modified Delphi method with the CAB was used to identify implementation outcomes to use in later steps of IM. An anonymous online survey of a validated community engagement measure assessed CAB members’ satisfaction with the process.

**Results:**

Using a modified Delphi method as part of the needs assessment of IM, the CAB identified three broad categories of strategies: Changing infrastructure using patient navigation; training and educating patients, navigators and providers; and supporting clinicians in case identification and management. Self-reported satisfaction with the IM and Delphi process was high.

**Conclusions:**

Implementation Mapping facilitated the use of available evidence, new data, and community engagement to identify strategies to improve the delivery of programs to reduce hereditary cancer disparities. The modified Delphi method was easy to administer in a virtual environment and may be a useful for others in community-engaged research.

## Introduction

There are over 50 recognized hereditary cancer syndromes, yet the appropriate identification and management of individuals with hereditary cancers remains a severely underutilized evidence-based cancer prevention and control strategy. Hereditary cancer syndromes put individuals at- risk for early age at diagnosis and a greatly increased lifetime risk of cancer. For example, the lifetime risk of breast cancer is more than five times higher among women with hereditary breast and ovarian cancer syndrome (HBOC), and the risk of ovarian cancer is 39/100 vs. 1/100 compared to the general population [1]. Individuals with genetic mutations associated with Lynch Syndrome have a lifetime risk of colon cancer that is 10 times higher than the general population [1]. Knowledge about one’s genetic risk status can help guide decisions related to cancer prevention and control behaviors such as early and more frequent screenings for cancers, risk reduction strategies such as preventive surgeries and/or medications to reduce circulating hormones, and even impact family planning decisions [2, 3]. In fact, the Cancer Moonshot (2016) identified cancer prevention and early detection approaches for individuals at high-risk for cancer as a key focus, especially research that seeks to increase the use of genetic testing and appropriate clinical management strategies [4].

Screening for HBOC, endorsed by the US Preventive Services Task Force since 2005 and a covered service under the Affordable Care Act, still suffers from disparate utilization with under resourced and racial and ethnic minority women less likely to be screened as compared to White and higher income women [6]. Failure to ensure adequate representation in genetic testing leads to differentially poor outcomes in individuals and their families and additional downstream effects that can amplify cancer disparities throughout the population. When the people who participate in genetic testing are not representative of population risk, the ability to link specific genetic changes to cancer risk is lower in the groups who are not represented. While genetic differences do not directly account for the well-documented racial and ethnic disparities in cancer burden, available information suggests that racial/ethnic differences in genetic risk assessment, counseling, and testing are widespread [5]. For example, some studies have shown that only one-third to one-half of racial and ethnic minority individuals with a suggestive family history of cancer have received genetic testing as compared to White individuals [5]. Therefore, the science will be advanced for some but not all groups, further widening the potential for disparities [7].

As new hereditary cancer risk discoveries are occurring at a rapid pace with the advent of personalized medicine and genomics, it is especially important that evidence-informed, culturally competent implementation strategies are put into place to ensure these discoveries are not only translated effectively but equitably. Failure to do so could exacerbate existing disparities and potentially create new ones. For example, as technology advances there will also be advances in the barriers of equitable implementation due to disparities in access [7]. Although research on implementation strategies for the management of hereditary cancer syndromes is relatively novel, available research indicates that patient and provider education and mass media campaigns about genetics have been successful at changing cancer genetics-related knowledge, attitudes, and self-efficacy [8–13]. Research focused on changes in clinical decision supports and informatics in primary care settings has demonstrated moderate improvements in the identification and management of individuals at high-risk for cancer [14]. Though not widely implemented, outreach to third party payers and integration with federally funded screening programs (i.e., the CDC’s Breast and Cervical Cancer Prevention Program) have also been shown to have potential for positive impact [7].

Patient navigation within and across healthcare systems has shown particular promise for addressing the complex barriers associated with identification and clinical management related to hereditary cancer care [15–20]. The barriers associated with uptake and delivery are numerous including lack of communication between patients and providers, communication provider to provider, fear about discrimination, cost, psychological distress, lack of decision supports especially in primary care settings, and limited system capacity and networks for genetic counseling and testing services [21–30].

Policy makers, researchers, and experts in genetics and genomics have called for more attention to public health implementation strategies for hereditary cancer, noting the need for integrated multi-level, multi-component approaches which have not historically been applied to this issue [4, 31, 32]. Notably, community engagement is central to both these approaches, but has not had explicit emphasis in the published literature. A foundational principle of Implementation Mapping (IM), a process for designing or tailoring implementation strategies, is community engagement in designing solutions [33]. The process of IM includes conducting a needs assessment, defining program level outcomes and logic model of change, identifying an implementation strategy, producing implementation materials, and evaluating outcomes. With each step, previous steps should be reviewed to ensure all objectives are addressed [33]. While IM is being increasingly applied, it has not yet been used to design multi-level implementation strategies for delivery of interventions related to hereditary cancer. Thus, this paper will address an important gap in the literature by demonstrating the use of a community-engaged process to identify implementation strategies for the detection and management of individuals at high-risk for cancer based on family history and hereditary cancer syndromes.

## Methods

Implementation Mapping (IM) [33] was used in the current project to design implementation strategies to improve the adoption, implementation, and maintenance of evidence-based strategies for the management of individuals at high-risk for cancer based on family history. This paper describes the activities (and especially the community engagement activities) conducted during the first three steps of the IM process: (1) conduct an implementation needs assessment and identify program adopters and implementers, (2) identification of the program outcomes, performance objectives, determinants, and targets for change, and (3) identification and selection of theory and evidence-based implementation support strategies [33]. The IRB of Colorado determined that the study did not need ethical approval. The project was determined exempt from IRB review by the Colorado Multiple Institutional Review Board (Study ID: 20-1911).

Community engagement is a critical component of the IM planning process in order to complete an accurate needs assessment, ensure barriers are addressed, and verify that implementation strategies are relevant and accessible to the community. Hereditary cancer, in particular, can benefit from community-engaged approaches due to the sensitive nature of disclosures and high level of health literacy and trust required to achieve equitable implementation and uptake [34]. As a result, a Community Advisory Board (CAB) was formed during Year 1 of the project (Cancer RESULTS—Resources, Engagement and Support for Use of Lifetime Tailored cancer prevention and control Services).

### Community Advisory Board formation and meetings

A Colorado statewide CAB was formed in June 2020. Composition of the group was pre-determined by the investigator team, as part of the Cancer Prevention and Control Research Network (CPCRN) in partnership with grassroots community-based organizations to represent various settings, populations, geographic areas, and roles. It comprised members from healthcare systems and community-based organizations that serve individuals who are medically under resourced in Colorado including rural areas. Potential members that fit this composition were identified by the community partner organization to participate in interviews, confirm their interest and discuss the time commitment and expectations for participation. A signed memorandum of understanding was then issued. Roles of participating members included healthcare providers including mid-level providers, patient navigators/community health workers from the community, public health professionals, third party payers and accountable care organizations, cancer survivors, and a licensed genetic counselor as a topic expert. The CAB was co-created and co-managed with a community-based organization, and members of the CAB were compensated following recruitment. Categories of participants included key partners who represent potential adopters, implementers, and disseminators of the identified intervention strategies prioritized through participation in the CAB. Based on the distribution of the CAB over a wide geographic region and due to considerations related to COVID-19, all CAB meetings and investigator team meetings were conducted via Zoom.

### Step 1. Conduct an implementation needs assessment using a Community Advisory Board

**Fig. 1:**
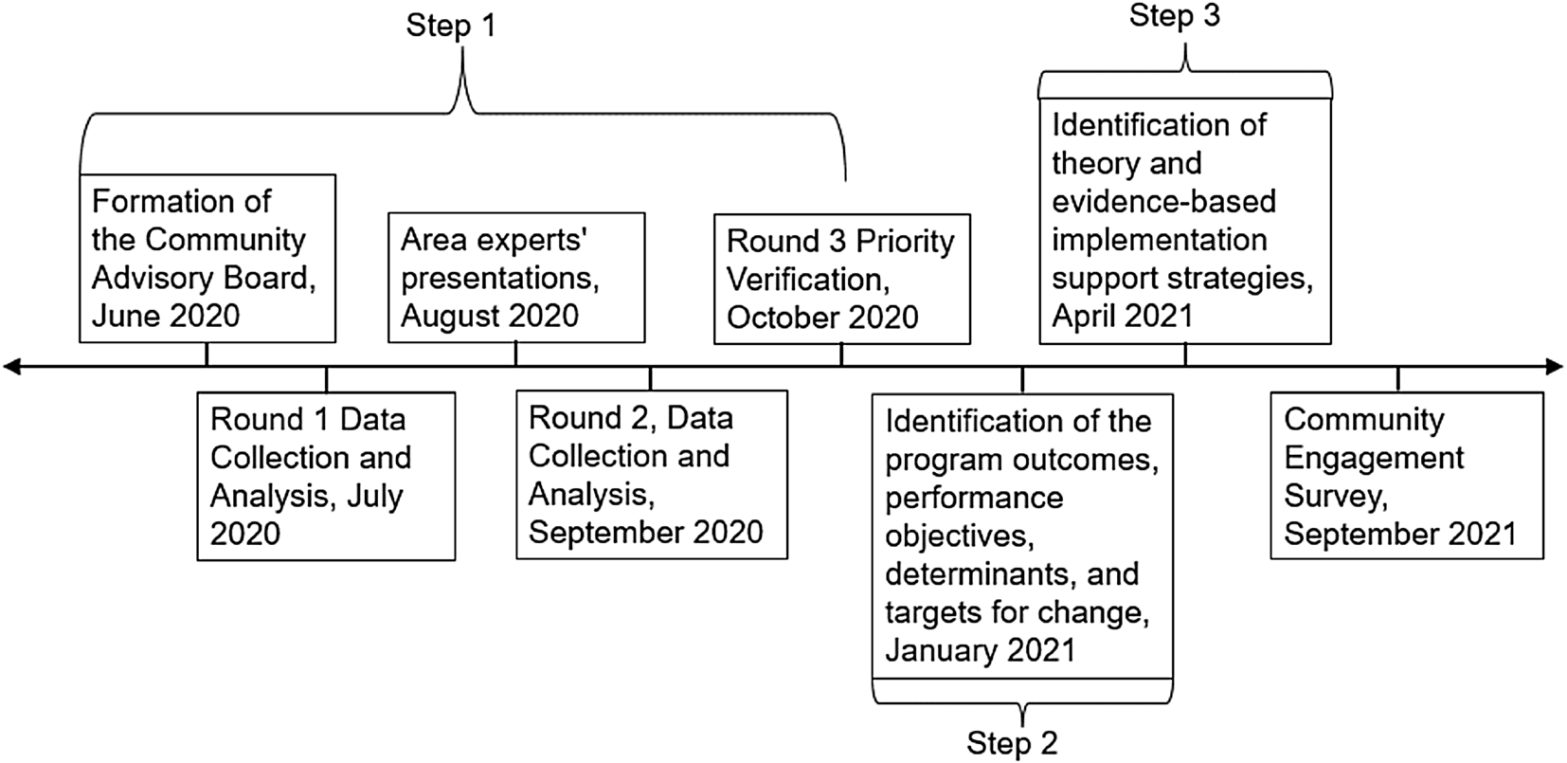
Timeline of Implementation Mapping (IM): Activities in Step 1–3 with Community Advisory Board (CAB)

Implementation needs assessment agendas were carefully constructed by both the investigator team and CAB partners. CAB meetings lasted 90 minutes, with 30–60 minutes per meeting dedicated for discussion and collection of feedback. Attendees were able to provide input through multiple methods (Qualtrics questionnaires, in-meeting polls, small, and large group discussions). Prompts, including open-ended questions, opportunities to provide feedback, and approval requests comprised the interactive meeting activities and members were intentionally assigned to specific discussion groups based on the area of need so that it was clear how their input was needed and would be used (i.e., expertise in hereditary cancer, healthcare delivery, public health, communication, etc.). Information and surveys were sent to members during the months where meetings were not scheduled. Frequency of meetings varied from monthly to quarterly over 2 years. The timeline of the CAB meetings can be reviewed in Fig. 1.

#### Modified Delphi method for conducting the needs assessment

**Fig. 2:**
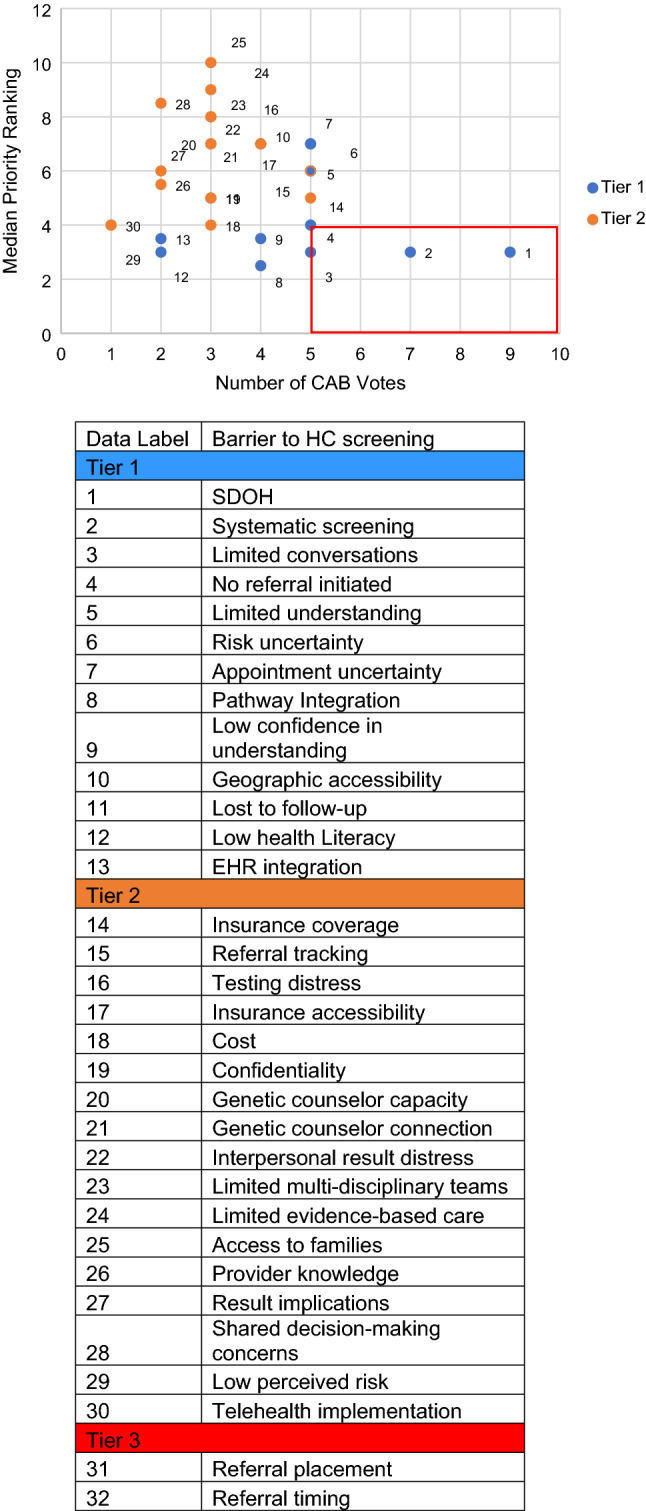
Final Delphi Ranking of Factors that Affect Hereditary Cancer Screening: Relative Rankings and Top Four Priorities. Items are ranked by lowest median score on a scale from 1 to 10 and by frequency of votes an item received. Tier 1 represented items with at least 4 votes, a median score below 4, and items that were emphasized to include during discussions and feedback from the steering committee. Tier 2 included items with at least 1 vote and median priority score of four or greater. Items in Tier 3 are not depicted in the figure because they were not considered in future steps. The four items in the red box are the final 4 priority items considered in step 1 of the needs assessment of IM

A modified Delphi method was utilized to execute the prioritization of needs related to individuals at high-risk for cancer based on family history. The Delphi method is a structured, iterative method of gathering and analyzing expert consensus to inform decision making and has been widely used in public health [35]. The investigators synthesized epidemiologic evidence to identify barriers to hereditary cancer screening, potential root causes, and the evidence for interventional approaches that could be supported through program activities, (see figure 2 for a list of these factors). The evidence review was presented to the CAB to inform the basis for the priority setting through iterative discussion and rounds of anonymous voting. Through three modified Delphi rounds, the CAB ranked priorities for collective action based on significance and potential changeability of the factor in their settings and community.

#### Delphi round 1 data collection and analysis

In the first round, CAB members ranked their perceived top priorities to address in future interventions, perceived changeability, and perceived feasibility of changing these priorities using an anonymous Qualtrics web-based survey [36]. The participants were asked to rank their top 10 priorities, with the lowest ranking score indicating high importance, and to rank the changeability and feasibility of each on a four-point Likert scale (not at all, somewhat, quite a bit, very changeable or feasible). Scores were evaluated to determine if mean or median values were most appropriate to report based on the distribution of scores.

Between Delphi rounds 1 and 2, as part of the Delphi process and at the request of CAB members, area experts gave presentations to provide the CAB members with additional information based on CAB meeting discussions in order to finalize the top priority areas and prepare for round 2.

#### Delphi round 2, data collection and analysis

The second round asked respondents to confirm the priorities identified by the synthesized literature, area experts, and the CAB members. Priorities were then organized into multi-level factors  of influence with a focus on the provider and system level. Scoring options, procedures and analysis were similar to those in round 1.

#### Delphi round 3, priority verification

Items from round 2 were included only if they had a better (i.e., lower) median priority score of four along with a changeability score of two or higher. Round 3, the final step in the process, was a live, web-based meeting with CAB members to confirm consensus from the results of round 2 on the final compilation of priorities. During the meeting CAB members were placed into groups to discuss the top priorities from the previous step. The end of the session called for a vote using in-meeting polling to confirm CAB’s acknowledged top priorities as a result of the needs assessment portion of IM.

### Step 2. Identification of the program outcomes, performance objectives, determinants, and targets for change

Utilizing the priorities identified in step 1 in modified Delphi method, multi-level performance objectives (or steps toward addressing the priorities) were drafted by the investigator team. Determinants were assessed by the investigator team through the review of health behavior theory and literature on hereditary cancer interventions to identify levers for behavior change at each level. For example, determinants included knowledge, self-efficacy, perceived advantage and other constructs from health behavior and dissemination theories. Other determinants such as lack of follow-up resources and communication were identified from reports of interventions in the literature. Change objectives were compiled into a change matrix (i.e., tables using determinants and performance objectives to create change objectives; see also Table 1) which were vetted with the CAB members to assess agreement and identify determinants that may have been overlooked. The CAB members were divided into groups to discuss and review program objectives at each level to obtain feedback. After the meeting, the suggestions were incorporated into each matrix by the investigator team.

**Table 1:** Partial change matrix for providers (implementers) of hereditary cancer screening in clinic setting displaying the use of determinants and performance objectives to create change objectives

Performance objectives/determinants	Knowledge	Skills	Confidence/self-efficacy (1)
Providers will counsel and screen patients for family history of cancer	Describe the role of family history on cancer risk List what to collect and from whom Identify recommendations for care Explain benefits of screening on patient care	Demonstrate skill to collect an accurate history Demonstrate ability to Interpret family history information	Have confidence they can identify and assemble internal and external resources for clinic to establish patient care pathways for family history screening and management
Providers will use results of family history to make appropriate referrals	Identify referral resources in their area Describe steps in care pathway for referrals	Demonstrate ability to initiate referral for genetic counseling and testing	Have confidence that they can arrange assistance to address barriers to care and ensure follow-up

Informed by the Consolidated Framework for Implementation Research, Inner and Outer Setting (https://www.cfirguide.org)

### Step 3. Identification of theory and evidence-based implementation support strategies

Based on the change objectives identified in step 2 of IM, corresponding evidence-based implementation approaches were reviewed. Literature review, presentations from area experts, and feedback from the CAB were used to identify evidence-based implementation approaches. Change objectives were mapped to evidence-based implementation approaches to determine which approach would address the top priority areas for improvement of hereditary cancer screening rates. The final mapped results were vetted to the CAB.

### CAB community engagement survey

CAB members completed a community engagement survey to assess and reflect on the process of engagement with investigator team during the first three steps of IM [37]. The survey included 11 engagement principles from Goodman et al. measuring CAB members role in their organization, how well they believe the investigator team exemplified one of the principles, and how often they thought the investigator team exemplified one of the principles. Each question was measured on a 5-point Likert -scale. The survey was administered anonymously using Qualtrics [36] 

## Results

### Community Advisory Board

The 22-member CAB established with the community-based organization included cancer program specialists, medical oncologists, program managers, genetic counselors, family physicians, directors of public or community health programs, and cancer survivor advocates representing urban, rural and frontier counties. Members of the steering committee identified their role in implementation as 1 (4%) funder, 1 (4%) put in place training, policies, and procedures to support implementation, 2 (9%) implement with others as part of a team, 4 (18%) advocates within their own organization, 2 (9%) advocate outside of their organization, 1 (4%) disseminate program, 6 (27%) investigator team, and 5 (22%) missing or did not answer**.** All but one member was retained across the first two years of the CAB.

### Step 1. Conduct an implementation needs assessment using a Community Advisory Board

#### Delphi round 1 survey and results

Delphi Round 1 of the survey included 32 items (Fig. 2) that affect hereditary cancer screening as identified through synthesized epidemiological evidence and literature review, which was presented to the CAB. These 32 items included factors related to: screening/collecting family history/case identification, psychosocial issues/perceived risk, genetic counseling, referral/referral tracking, integration/continuity of care, disparities/barriers/access. Delphi round 1 was sent via email to 18 individuals, of which 11 (61.1%) CAB members completed the survey.

Results of Delphi round 1 were categorized into three tiers based on the distribution of observed values: Tier 1 (*n* = 13) represented items with at least four votes, a median priority score below four (lower score = higher priority). Tier 2 (*n* = 17) included items with at least one vote and median priority score of four or greater. Tier 3 (*n* = 2) included items that did not receive any votes and were therefore excluded in future rounds of voting. The list and results of the 30 remaining items and the Delphi round 3 are also shown in Figure 2*.* The top four priorities with the highest number of votes and the lower median scores (higher priority) are shown in the bottom right of the graph (red box)*.*

Overall, in round 1, the mean impact score was 3.55 on a 4-point Likert scale, with 4.00 indicating that the respondent was quite a bit confident that the determinants would have a significant impact on hereditary cancer screening.

Between rounds 1 and round 2, at the CAB request, three presentations from subject matter experts focused on access and coverage of insurance for genetic testing, organizations workflow with working with other providers outside their system, and patient navigation to address social determinants of health.

#### Delphi round 2 survey and round 3 discussion results

Where items appeared to overlap, the top factors from Tiers 1 and 2 were combined and vetted with the CAB members to create a list of 11 priorities for round 2 to reflect multi-level priorities at the patient, provider, and system level.

Rounds 2 and 3 resulted in the identification of four top priorities where CAB members felt change could be made to increase hereditary cancer screening. The items include (1) patients experience barriers to care related to hereditary cancer, including barriers to genetic counseling such as insurability, access to care, health literacy, and social determinants of health (SDOH), (2) lack of access to services due to distance, transportation, cost concerns, referral barriers related to social determinants, and information about availability of services/clinics (systematic screening), (3) lack of system-level supports to address barriers to care and ensure care pathway is followed (e.g., patient navigator or community health worker) (limited conversations), and (4) lack of systematic way to identify patients and family members who could benefit from genetic counseling and testing (no referral initiated) Fig. 2. In round 2, the mean impact score was 3.17 on a 4-point Likert scale, with 4 indicating the respondent was ‘quite a bit’ confident that the determinants would have a significant impact on hereditary cancer screening.

### Step 2 and 3: Mapping of program objectives and corresponding evidence

**Fig. 3:**
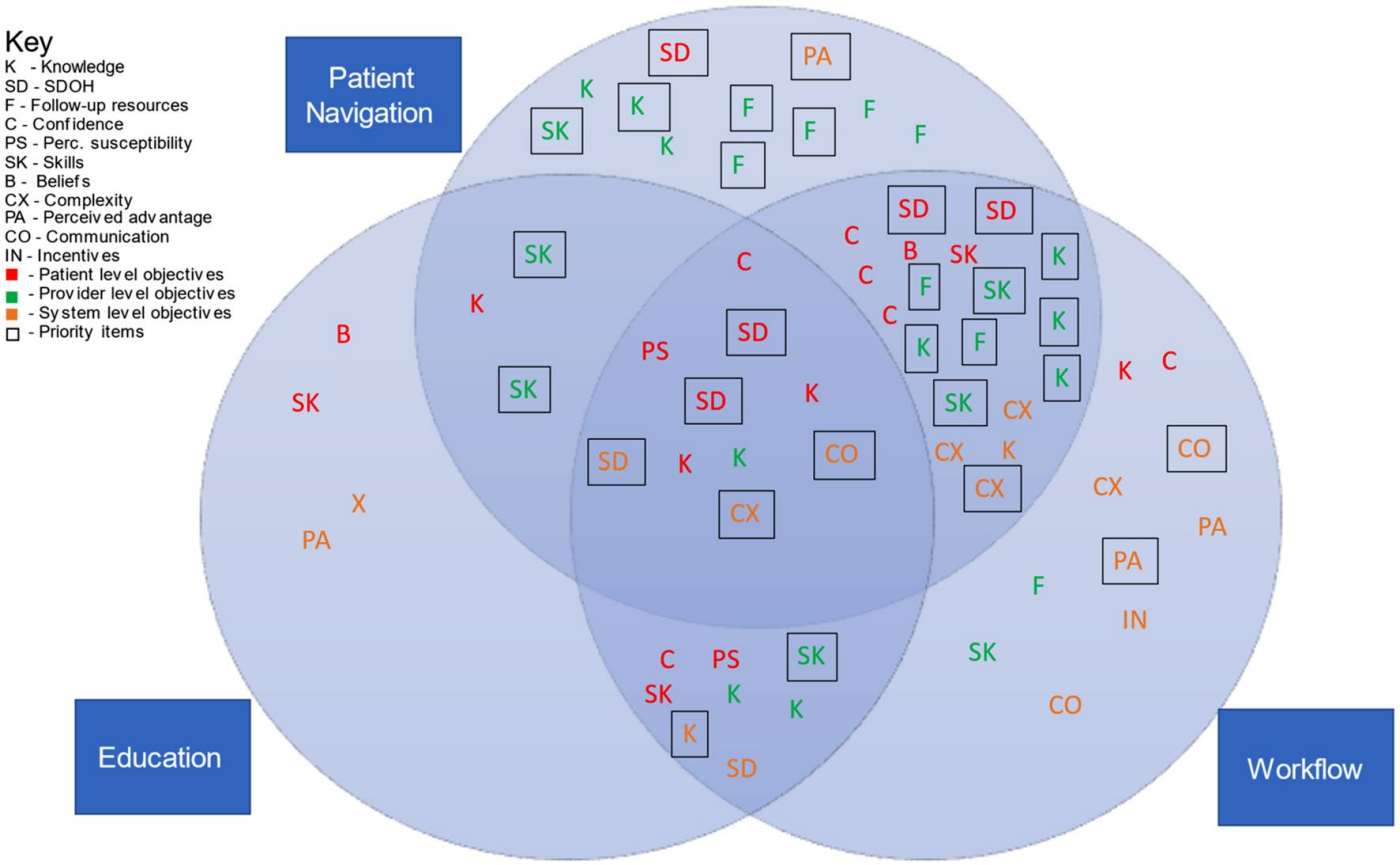
Implementation mapping of change objectives matched to evidence-based approaches. The figure aims to depict the mapping process and how change objectives were mapped to evidence-based implementation approaches. Statistics provided within the results section

A multi-level change matrix identifying performance objectives and determinants at the patient, provider, and system level was developed by the investigator team and vetted with the CAB (see Table 1 for an example of a partial matrix for providers; see Fig. 3 for a full list of determinants). The change objectives were mapped onto evidence-based implementation support strategies, similar to the approach of Walker et al. [38]. Evidence-based implementation strategies identified included patient navigation, patient and provider education, and workflow/system changes. A total of 65 change objectives, including 29 that mapped to high priority areas from the above process, were mapped to these evidence-based implementation strategies, indicating good concordance. More specifically, patient navigation mapped to 43 (66.2%) change objectives including 25 (86.2%) priority objectives; education mapped to 25 (38.5%) change objectives including 9 (31.0%) priority objectives; and workflow resources mapped to 47 (72.3%) change objectives including 20 (69.0%) priority items Fig. 3. Although the focus of the planning and prioritization was on factors related to implementation, the CAB requested to include factors at the patient, provider, and system level during this step to facilitate conceptualization of the overall program and goals.

The mapping of change objectives was reviewed and approved by the CAB, indicating that the approaches address the previously established priority areas. The total process including the evidence review and Delphi process took 9 months Fig. 1*.*

### CAB Community engagement survey results

The community engagement survey which allowed for anonymous responses was administered using Qualtrics and was completed by 9 of 17 (53%) CAB members. Overall, CAB members reported high satisfaction with the investigator team. Areas of strength included “Show appreciation for the CPCRN Steering Committee’s time and effort”; “Value the CPCRN Steering Committee perspectives”; “Show appreciation for the CPCRN Steering Committee’s time and effort”; “Value the CPCRN Steering Committee perspectives”. For these items the CAB members indicated “Excellent” or “Always” 89% of the time or greater.

Areas of growth indicated by the CAB members included, “Involve CPCRN Steering Committee members in sharing health messages in community settings” (*n* = 2), “Make plans for CPCRN Steering Committee activities to continue for many years” (*n* = 2), “Want to work with CPCRN Steering Committee members for many years” (*n* = 1). These items were indicated to as “fair” or “rarely” in exemplifying the specific principles.

## Discussion

Overall, using IM to conduct a needs assessment, and identify theory and evidence-based implementation support strategies through a community-engaged process helped identify implementation strategies for the detection and management of individuals at high-risk for cancer based on family history and hereditary cancer syndromes. Using IM method (as described in step 1) supported by a modified Delphi method helped the CAB identify the needs of their community at multiple levels, recognize and select implementation strategies based on existing evidence, and identify capacity building activities in support of these strategies to increase the rate of hereditary cancer screening in their community.

The recruitment of diverse roles and positions across the healthcare system and community allowed for community-engaged efforts to be representative of hereditary cancer screening across the care experience, from primary care, genetic counseling, insurance coverage, community health workers, and the cancer survivor perspective. Throughout the utilization of IM, the CAB encouraged reflection and expansion of key issues to reflect the needs of the community within the present time. The engagement with the CAB provided a multi-level approach that emphasized four priority areas that without engagement of the CAB may not have been addressed. For example, many of the existing interventions focus on education of primary care providers and information technology supports [8–14] yet these did not emerge as a top priority of the CAB in the Delphi process. Notably, SDOH emerged as top tier factor even in the first round of the Delphi (Fig. 2), indicating the importance of addressing disparities among CAB members. As such, the strategy of patient navigation was identified as a promising intervention for the CAB to support based on both evidence of its potential impact across patients, providers and the system as well as its ability to reduce barriers to care. This represents a potential evolution in strategies from those commonly delivered and reported in the literature and one that addresses disparities at its core.

The priority setting process and understanding existing evidence, guided by implementation mapping, were deemed highly valuable by the community members. The initial summary and presentation of evidence from literature for the needs assessment and identification of potential implementation strategies for the CAB was an in-depth process on the part of the investigator team, taking 3–4 months before deemed ready for presentation. Evidence was summarized into themes with a focus on implementation considerations rather than study design rigor (e.g., what settings and population were represented in the intervention, staffing and budget implications, with a general statement about strength of evidence rather than detailed description of methods). Notably, two meetings after subsequent discussion the CAB requested additional information on specific topics from this evidence review. This suggests that multiple opportunities for feedback over time be created in the meeting agendas, and that these opportunities for continued discussion are more important to CAB members than reviewing empirical evidence, which is often deemed most important by researchers. In discussions and post-meeting communications, CAB members indicated they were especially positive about the rating and ranking method from the Delphi process to narrow down and numerically identify potential priorities from the evidence review for group discussion. The CAB members also routinely expressed appreciation for the small group activities because it allowed them to connect and network with other members they may not interact with on a regular basis. Thus, this is an important value to consider when recruiting and establishing group norms for community advisory boards. The composition of these in-meeting small groups intentionally varies depending on the discussion item to allow sharing of ideas and perspectives across individuals with varying roles and responsibilities, which was also deemed a strength. Minutes from meetings were very helpful in assisting the investigator team with following up on discussion items, which were usually the first item on the agenda.

Through examination of the identified priorities through patient, provider, and system levels the results reflect the need for multi-level, multi-component approaches to hereditary cancer screening [7, 31]. Many existing interventions do not address this need and are instead focused on single levels or components. For example, while educational interventions have been shown to change knowledge and attitudes, there is little available research on whether or not this actually changes patient or provider behavior and what limited evidence does exist, suggests that it does not actually increase screening rates [9–12, 26, 40]. Similarly, interventions to improve clinical information systems and decision supports failed to find meaningful change in hereditary cancer care. As the CAB in the present study identifies the need for considering change on patient, provider, and system levels, future implementation protocols and materials may benefit from addressing change objectives from multiple levels.

Although reviewing the determinants gave the CAB members a good idea of target program components, this most likely could have been accomplished through a higher-level summary with the same result which would have shortened the process. Another area of improvement, which was noted in the survey results from the CAB, is that they were looking for ways to bring information back from the process to their settings. In the future, working earlier with community on dissemination products will be an important aspect.

The implementation strategies (step 3) of patient navigation, education, and system workflow changes identified in the literature and from the CAB fell into three categories recognized by *ERIC* (Expert Recommendations for Implementing Change Project): *Changing infrastructure* using patient navigation; *training and educating* patients, navigators, and providers; and *supporting clinicians* in case identification and management [39]. Thus, although the ERIC were not used a priori to identify implementation support strategies, the implementation strategies identified through IM by the CAB are concordant with current implementation science theory which suggests a high likelihood of success.

The intersection of implementation science and personalized medicine points to the importance of a deep understanding of contextual issues in addressing the need for hereditary cancer screening [31]. Combined with the lack of meaningful improvements observed with existing single target interventions, this calls for engagement of communities to help develop new strategies and ensure that they are feasible and meaningful. The current project demonstrated that IM effectively engaged community, demonstrated in the high scores of the community engagment results, and resulted in identification of a multi-level implementation support strategy that combined components of patient navigation, education, and workflow in ways not previously reported in the literature. More specifically, the multiple program components identified by the CAB include an educational component for training of navigators and team members, barrier reduction through patient navigation, and support for providers in the systematic identification and management of patients through workflows that include patient navigation to promote coordination and communication. As shown in Fig. 3, patient navigation mapped to multiple areas identified by the CAB as well as the ones deemed highest priority.

Limitations of this study include small numbers of individuals on the CAB, and recognition that members may not represent all the viewpoints from the communities and organizations they serve. Additionally, these results are a blueprint for subsequent programmatic activities and therefore the true value of the planning process in determining the reach and impact of the strategies identified is currently untested. Next steps from these findings are to use the results to guide capacity building and the development of implementation supports in the designated three focus areas (Fig. 3). Experience of other researchers in the CPCRN indicates that mini-grants, the provision of technical assistance, and trainings are commonly used successfully to address complex prevention and control programs [41–43]. Many additional potential opportunities to improve hereditary cancer screening and management exist but require more extensive resources and coordination than possible with this project. This includes potential integration of screening for family history in the National Breast and Cervical Cancer Early Detection Program (NBCCEDP) and the Comprehensive Colorectal Cancer Screening Programs (CCSP). Additionally, educational and other resources that are not focused on specific cancers, but multiple organs are needed, since hereditary syndromes can cause many forms of cancer. For example, the Facing Our Risk of Cancer Empowered (FORCE) is one of the few organizations to recently expand their focus to include multiple cancers, not just breast, ovarian or Lynch. In alignment with the goals of the Cancer Moonshot [4], the coordination of activities across multiple partners with engagement of the community has the potential to ensure that new discoveries in genetics and genomics will reduce rather than exacerbate cancer-related disparities.

## Data Availability

The datasets generated during and/or analyzed during the current study are not publicly available due to small sample sizes and concerns regarding confidentiality but are available from the corresponding author on reasonable request.
